# Extreme Longevity: Analysis of the Direct or Indirect Influence of Environmental Factors on Old, Nonagenarians, and Centenarians in Cilento, Italy

**DOI:** 10.3390/ijerph19031589

**Published:** 2022-01-30

**Authors:** Silvana Mirella Aliberti, Francesco De Caro, Richard H. W. Funk, Luigi Schiavo, Joseph Gonnella, Giovanni Boccia, Mario Capunzo

**Affiliations:** 1 Department of Medicine, Surgery and Dentistry “Scuola Medica Salernitana”, University of Salerno, 84081 Salerno, Italy; fdecaro@unisa.it (F.D.C.); lschiavo@unisa.it (L.S.); gboccia@unisa.it (G.B.); mcapunzo@unisa.it (M.C.); 2 Medical Faculty Carl Gustav Carus, Institute of Anatomy, University of Technology (TU) Dresden, 01307 Dresden, Germany; Richard.Funk@di-uni.de; 3 Center for Medical Research in Medical Education and Health Care, Sidney Kimmel Medical College, Thomas Jefferson University, Philadelphia, PA 19107, USA; joseph.gonnella@jefferson.edu

**Keywords:** old population, centenarians, longevity, environmental factors, healthy living, cilento region

## Abstract

Longevity is rightly considered one of the greatest achievements of modern society, an achievement understood as the possibility of increasing the healthy part of life and not only its full duration. This study investigated the phenomenon of regional longevity in Cilento by analyzing the association between longevity indicators and some environmental factors, in order to understand if factors such as altitude, climate, UNESCO protected areas, and hinterland can directly or indirectly influence the measure of healthy living and lead to longevity. Demographic and environmental data were collected through the Archives of the Cilento municipalities, the National Institute of Statistics, the Italian Institute for Environmental Protection and Research, and the Italian National Commission for UNESCO. The Geographically Weighted Regression were used to determine the association between longevity indicators and environmental factors. Correlation analysis between the longevity indicators was investigated in order to have a complete picture of longevity in Cilento. It was discovered that Cilento longevity is mainly found in the central area of the territory and from there, by age groups, it widens towards the south-eastern area. This designated area is part of the hilly area of the Cilento, at an altitude between 400 and 700 m above sea level. The towns of this area are part of the UNESCO heritage and are characterized by a transitional climate between Mediterranean and temperate climate. Moreover, the correlation analysis between the six indicators of longevity has confirmed a linear relationship between the six variables and this indicates that in the years to come there will be the probabilities of a generational turn over between the old, great old, nonagenarians and current centenarians, provided that the SARS-CoV-2 pandemic subsides.

## 1. Introduction

Due to the decline in birth rate and the increase in life expectancy, the population worldwide is aging at an unprecedented speed. According to data from World Population Prospects [1], there are 727 million older people (aged 65 and older) and half a million centenarians (aged 100 and older) in the world and this trend will tend to increase in the future, provided that the SARS-CoV-2 pandemic subsides [2,3]. According to some studies, in fact, there is a greater vulnerability of the elderly but also a resilience of centenarians [4,5].

Longevity is rightly considered one of the greatest achievements of modern society. On the topic of longevity and aging, it is important to make a distinction between old, great old, nonagenarians, and centenarians. According to the current literature, the population over sixty-five years old is defined as old, the great old population is composed of those over eighty-five years, nonagenarians are represented by the population over ninety years and centenarians are the population that are from a hundred to more than a hundred years. Longevity indicators are usually used to define the difference, although the literature is currently controversial on the topic. In agreement with several authors to measure old age the Aging tendency is used [6], for the great old the 85+ ratio, for nonagenarians the 90+ ratio [7], and for centenarians the centenarian prevalence [8], which reflects the extreme longevity of a region’s total population and the proportion of the population that is aging, so that comparisons can be made easily across regions. The Centenarity index and the Longevity index were introduced to reduce the effect of phenomena such as the decline in the birth rate and migration observed in regional territories [7,9].

Among the myriad of genetic and behavioral factors, it is important to understand which factors are capable of modifying the epigenome to establish healthy aging. Epigenetics [10,11,12] may play a key role in providing answers to this question. In humans, factors that can influence epigenetic status can be divided into: environmental, nutritional, medical care, and lifestyle. 

Longevity has been associated with specific lifestyles, such as the tendency to avoid conflict situations, the ability to respond positively to stress [13], attachment to family and religion [14], and also individual personality factors [15]. Healthy nutrition and moderate physical activity are also universally accepted to be key components in preventing the processes that promote aging [16,17]. 

Kaplanis et al. [18] stated that analysis of family trees with millions of relatives shows that heritability probably does not exceed 16%, while Ruby et al. [19] pointed out that due to assorted mating, the true genetic component of longevity, widely overestimated in the past, is probably less than 10%. Other studies have found that the heritable component of human longevity has been estimated to be between 15 and 40% [20,21,22,23,24,25,26]. A study of Scandinavian twins [27] concludes that the effect of genetic inheritance on life expectancy accounts for 20–30%, while that of environmental changes accounts for at least 70%. 

The environment has a large effect on the longevity of regional populations as it is able to modify the epigenome to establish a “healthy” transcriptome [12]. 

Several studies have shown that altitude, soil type, and crop agrotype [28,29,30] are the main environmental factors that influence the longevity of regional populations. 

The effects of climate, including temperature, air pressure, precipitation, and sunshine hours, on human health and lifespan are also well documented. According to Li et al. [31], 90-year mortality is closely related to climate change. While Cheng [32] found that the regions with the longest-lived people, all have an average temperature of about 20°. Other studies have shown that the high percentage of long-lived people in Georgia is the combined result of climate, topography, and other ecological factors [33]. Robine et al. [34] confirmed that an important role in longevity may be played by the mean temperature of 17.2 °C. 

Under particularly favorable environmental conditions, some groups of humans demonstrate the ability to reach and exceed 100 years of age, in fair health [10,11,12].

There are several populations that are among the longest lived in the world, including: the residents of the Japanese island of Okinawa, the Nicoya Peninsula in Costa Rica, Loma Linda in California, Icaria in Greece, and the centenarians of the province of Ogliastra in Sardinia, Italy. Residents of these areas are among the regions defined by researchers as “Blue Zones” [35,36,37]. Several researches in the Blue Zones, highlight lifestyle and diet as peculiarities of long life [34,36,38,39]. 

There are also other regions in Italy, besides Sardinia, where there is a high prevalence of nonagenarians and centenarians [40,41,42,43]. One example is Cilento which was analyzed in this study. Several recent researches have dealt with longevity in Cilento [14,15,44]. Our hypothesis is that some environmental indicators, such as altitude, climate, and rural hinterland may be protective factors for longevity. Therefore, the aim of the present study was to investigate the phenomenon of regional longevity in Cilento by analyzing the association between longevity indicators and some environmental factors, in order to understand if factors such as altitude, climate, UNESCO protected areas, and hinterland can directly or indirectly influence the measure of healthy living and lead to local longevity.

## 2. Materials and Methods

### 2.1. Description of the Case Study: The Cilento Region

**Figure 1:**
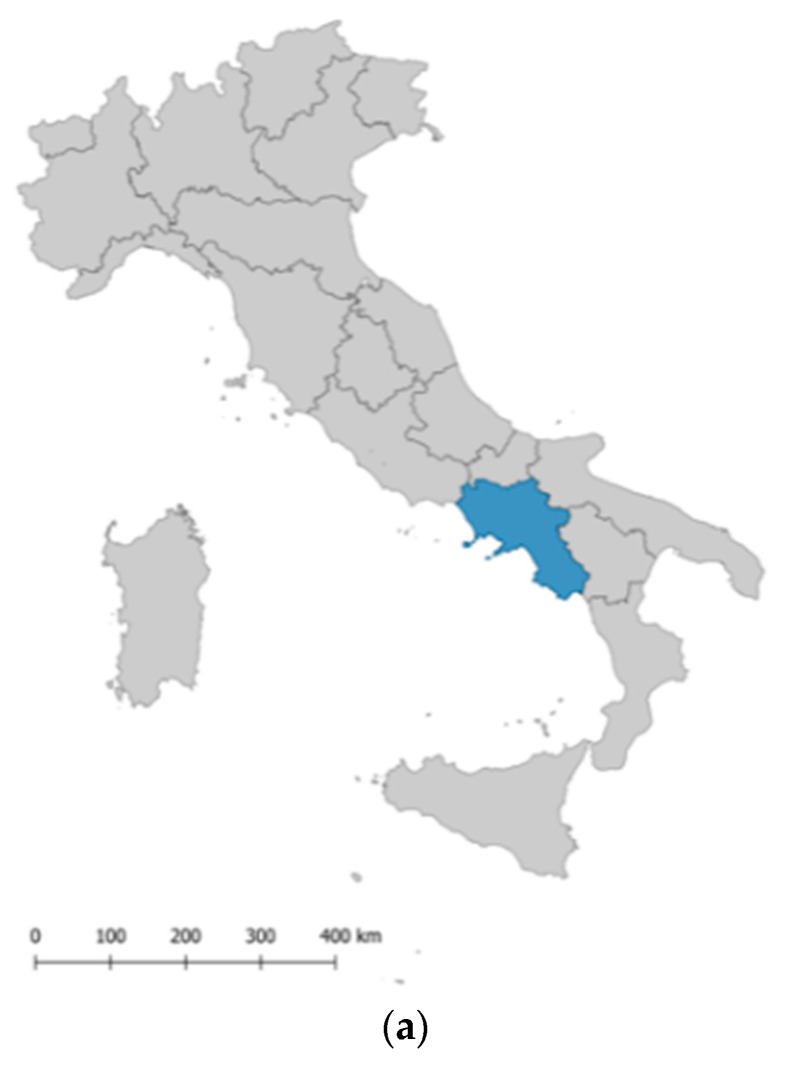

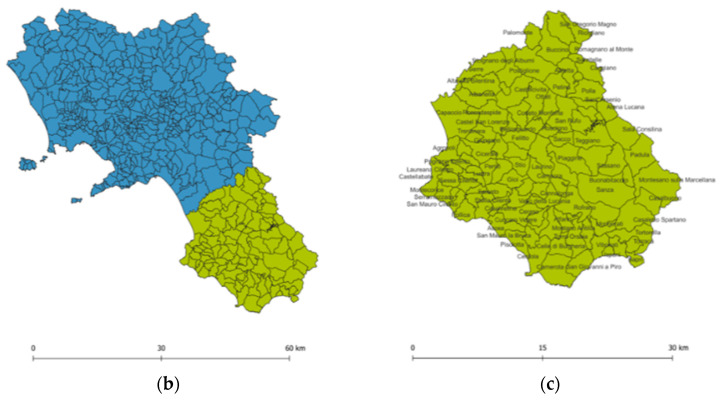
Maps for locating the study site. Notes: (**a**). Italy with the location of the region Campania; (**b**). the location of Cilento in the region Campania 1 (**c**). the territorial area of Cilento with the location of the 102 municipalities.

Cilento is an area located in the Campania region, in southern Italy. The territorial area of Cilento includes 102 towns (Figure 1), covering about 490,000 hectares. From the environmental point of view, it is a homogeneous territory, characterized by the integration of different landscape types including coastal, hilly, and mountainous areas, the mouths of important watercourses, and the geomorphological and climatic variability creates a potential not easily found in other areas of the Italian Peninsula. In this area, man has been able to integrate harmoniously with the forms of the landscape [45].

With a substantial old population base, Cilento is an ideal region to examine how regional longevity is associated with several natural factors.

### 2.2. Defining the Old Population

Cilento has a population of about 288,185 inhabitants and maintains relatively high proportions of long-lived people (old, nonagenarians, and centenarians) with a ratio of 43.05/1000 inhabitants [46]

In this study, six longevity indicators (Centenarian ratio, Aging tendency, 85+ ratio, 90+ ratio, Longevity index, and Centenarity index) were chosen to fully reflect the extreme regional longevity, the longevity of nonagenarians and centenarians within the population and the overall old population in Cilento. Two indices were used to reduce the effect of important phenomena such as declining birth rates and migration observed in regional territories: Centenarity index (CI%), given by the ratio of centenarians to those over 90, and Longevity index (LI%), given by the ratio of those over 90 to those over 65 [9]. The longevity indicators used are summarized in Table 1.

### 2.3. Environmental Variables Influencing the Structure of Old Age

Environmental factors were selected to understand their influence on regional longevity. The selection of these factors was based on review of relevant literature and data availability. The selected natural indicators include altitude, climate, UNESCO heritage area, and hinterland. Altitude was divided into plain, hill, and mountain based on meters of elevation, according to the altitude bands established by ISTAT [47]. For the climate of Cilento the mean annual temperature in degrees Celsius and the annual precipitation in millimeters (period of analysis year 2020) were analyzed [48], according to the climatic regions identified in Cilento by the directives of the Campania region [49]. In addition, the municipalities of Cilento were distinguished in number of municipalities present in UNESCO protected areas and municipalities located in areas defined as buffer zones because they are not included in the protected area [50]. Finally, the municipalities of Cilento were distinguished into municipalities present in the coastal area and municipalities in the hinterland [47]. These main factors and their subgroup sections are summarized in Table 2.

### 2.4. Data Source and Methods of Analysis

#### 2.4.1. Data Source

For this study, population data were collected through the Archives of the Cilento municipalities, in the years 2019–2020 and compared with ISTAT [51] (2020) population statistics. Longevity indicators were calculated from the collected population data and used as dependent variables in the study. Environmental indicators, used in the research as independent variables, were collected through: the database of the Italian National Institute of Statistics [47] for altitude and coastal/no coastal areas; data from the Italian Institute for Environmental Protection and Research [48] for climate; and the data from the Italian National Commission for UNESCO [50] for the towns of Cilento belonging to UNESCO.

#### 2.4.2. Statistical Analysis

This study used the STATA software [52] to calculate longevity indicators from the National Institute of Statistics 2020 and Cilento Municipal Archives 2019–2020. Correlations among the six longevity indicators were performed using STATA. QGIS 3.14.15 [53] was then used to produce spatial distribution maps of longevity and environmental variables. Multiple approaches were applied to address the research questions. Global Moran’s I index and local Moran’s I index (spatial autocorrelation analysis) were used to describe the characteristics of the global and local spatial cluster value of the longevity index, through ArcGIS [54]. An Ordinary Least Squares (OLS) regression and a Geographically Weighted Regression (GWR) model, with ArcGIS software, were used to study the spatial relationship between the explanatory variables and the longevity outcome variables in the given dataset. Finally, we compared the performance between the OLS regression and the GWR model. In the models presented, we eliminated variables that had a VIF value greater than 7.5 because GWR is sensitive to multicollinearity. 

##### Spatial Autocorrelation Analysis

Moran’s index (Moran’s I) was used to measure the spatial autocorrelation of the indicators and was calculated using the following formula [55]:

(1) $$I=\frac{n\sum_{i=1}^{n}\sum_{j=1}^{n}\omega_{ij}\left(x_{i}-\bar{x}\right)\left(x_{j}-\bar{x}\right)}{s_{0}\left(\sum_{i=1}^{n}\sum_{j=1}^{n}\omega_{ij}\right)\sum_{i=1}{\left(x_{i}-\bar{x}\right)}^{2}}$$

where *n* is the number of spatial units indexed by *i* and *j*; *x* is the variable of interest; *x_i_* and *x_j_* are the values of the observed variable at sites *i* and *j*; $\bar{x}$ is the mean of *x*; and the weights *w_ij_* are written in a (*n* × *n*) weight matrix. The weight matrix depicts the relationship between an element and its surrounding elements. Weight can be based on contiguity relationship or distance. The value of Moran’s I usually ranges from −1 to +1. A positive Moran’s I value denotes positive spatial autocorrelation, whereas a negative value denotes negative spatial autocorrelation. For statistical hypothesis testing, values of Moran’s I were evaluated based on Z-score. A |Z| value higher than 1.96 indicates significant spatial autocorrelation at the 0.05 significance level, and a |Z| value higher than 2.58 indicates significant spatial autocorrelation at the 0.01 significance level.

In this study, spatial autocorrelation analysis (Moran’s I) between the dependent variables, showed a value ranging from 0.08 to 0.35 and these variables had relatively positive Z and *p* values.

However, the global spatial autocorrelation index measures the overall spatial autocorrelation of our dataset and does not indicate where clusters of high or low longevity might occur. Therefore, local indicators of spatial autocorrelation are needed.

The local Moran’s I [56] equation is as follows:

(2) $$I_{i}=\frac{z_{i}-\bar{z}}{\sigma^{2}}\underset{j=1\;j\neq i}{\sum }\left[w_{ij}\left(z_{j}-\bar{z}\right)\right]$$

where *z_i_* is the value of the variable *z* at location *i*; *z* is the average value of *z* with the sample number of *n*; *z_j_* is the value of the variable *z* at all the other locations (where *j* ≠ *i*); σ^2^ is the variance of variable *z*; and *w_ij_* is a weight which can be defined as the inverse of the distance *d_ij_* among locations *i* and *j*. The weight *w_ij_* can also be determined using a distance band: samples within a distance band are given the same weight, while those outside the distance band are given the weight of 0.

A high positive local Moran’s I value implies that the location under study has similar high or low values to its neighbors, so the locations are spatial clusters. Spatial clusters include high-high clusters (high values in a high-value neighborhood) and low-low clusters (low values in a neighborhood.

A high negative local Moran’s I value means that the location under study is a spatial outlier. Spatial outliers are those values that are obviously different from the values of their surrounding localities. Spatial outliers include high-low (a high value in a low-value neighborhood) and low-high (a low value in a high-value neighborhood) [57].

##### OLS Regression and GWR Model

To quantify the impacts of environmental factors on longevity indicators, we used OLS regression, which is the most commonly used method for analyzing relationships between two or more variables. OLS is a traditional regression method that estimates a global regression coefficient, which is constant over space. The equation for OLS is as follows:

(3) $$y=\beta_{0}+\overset{p}{\underset{i=1}{\sum }}\beta_{i}x_{i}+\varepsilon$$

where *y* is the dependent variable, *β*_0_ is the intercept, *x_i_* is the *i*th independent variable, *β_i_* is the *i*th regression coefficient, *ε* is the error term, and *p* is the number of independent variables. OLS cannot test spatial effects between observations and does not take into account spatial autocorrelation, so we applied GWR to measure altitude, climate, UNESCO listed area or buffer zones, coastal and inland areas in terms of spatial impact.

GWR is an extension of OLS regression that allows locally varying parameters to consider a spatial nonstationary in a sample [58]. The GWR formula is as follows:

(4) $$y_{i}=\beta_{0}\left(u_{i},v_{i}\right)+\underset{k}{\sum }B_{k}\left(u_{i},v_{i}\right)x_{ik}+\varepsilon_{i}$$

where *y_i_* refers to the dependent variables such as Centenarian ratio, Aging tendency, 80+ ratio, 90+ ratio, Longevity index, and Centenarity index, at location *i*. (*u_i_*, *v_i_*) means the coordinates of the centroid at location *i*. *β*_0_(*u_i_*, *v_i_*) refers to the intercept for location *i*. *βk*(*u_i_*, *v_i_*) means the local parameter for independent variable *k* at location *i*. *x_ik_* is the value of independent variable *k* at location *i*. *ε_i_* is the error term for location *i*.

(5) $$\beta \left(u_{i},v_{i}\right)={\left(X^{T}W\left(u_{i},v_{i}\right)X\right)}^{-1}X^{T}W\left(u_{i},v_{i}\right)Y$$

where *β*(*u_i_*, *v_i_*) means the local regression coefficient at location *i*. *X* refers to the matrix of the independent variables of the environmental factors. *Y* is the vector of the dependent variable of longevity indicators. 

##### Comparison of OLS Regression and GWR Models 

We compared the model performance between the OLS regression and GWR models based on the coefficient of determination (R^2^) and corrected Akaike Information Criterion (AICc) values. The R^2^ presents the prediction ability of a regression model to fit the measured values of the dependent variable, and the AICc is an indicator of the relative information lost by the model during the estimation process [58]. 

## 3. Results

### 3.1. Evaluation of the Spatial Distribution of Longevity Indicators in Cilento, Italy

**Figure 2:**
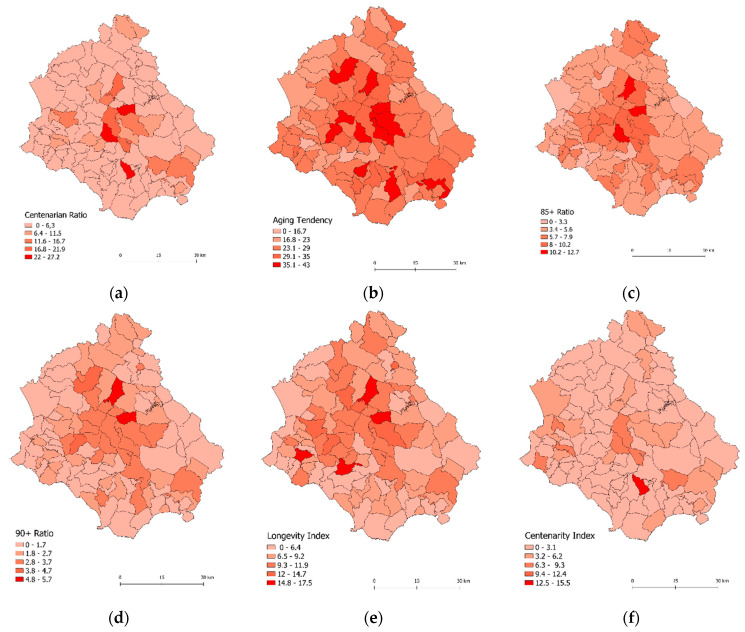
Spatial distribution of the six longevity indicators in Cilento. Note: (**a**) Spatial distribution of Centenarian ratio; (**b**) Spatial distribution of Aging tendency; (**c**) Spatial distribution of 85+ ratio; (**d**) Spatial distribution of 90+ ratio; (**e**) Spatial distribution of Longevity index; (**f**) Spatial distribution of Centenarity index.

The longevity area in Cilento is clearly delineated in the central municipalities of the territory (Figure 2) and from there, by age groups, it widens towards the south-eastern area. The towns with the highest Centenarian ratio (Figure 2a) are located in the central/central-eastern part of the region and are: Campora (27/10,000), Laurito (26/10,000), Sacco (22/10,000), Laurino (21/10,000), Piaggine (16/10,000).

Throughout Cilento the Aging tendency (Figure 2b) is very high, particularly in the following towns: Campora (42.1), Roccagloriosa (41.6), Valle dell’Angelo (40.4), Sacco (39.8), Sant’Angelo in Fasanella (38.7), Castelcivita (38.6), Piaggine (37.3), located in the central and southeastern areas of the region.

The ratio 85+ (Figure 2c) and 90+ (Figure 2d) show a similar longevity trend, with a higher concentration in the northern, central, and south-eastern part of Cilento.

Influenced by migration and birth rates, Aging tendency, 85+ ratio, 90+ ratio, and Centenarian ratio cannot accurately explain the regional distribution of longevity in Cilento. To exclude the influence of migration and birth rates, Centenarity index and Longevity index were introduced [9].

The highest Longevity index (Figure 2e) is held by the central and north-western areas of Cilento, and the reference towns are: Sant’Angelo a Fasanella (17.5), Sessa Cilento (17.4), Sacco (15.2), and Vallo della Lucania (14.7). 

Figure 2f shows the distribution of the Centenarity index, which is displaced in different areas of Cilento, with a higher index in the central, central-western, and south-eastern areas, among the reference towns: Lustra (9.09), Campora (9.09), Montecorice (8.84), Celle in Pittari (8.82), Laurino (7.69).

The six indicators analyzed all show high levels of longevity in the central and south-eastern area while they have lower levels towards the borders with Basilicata and the coastal zone.

Based on the results of the global autocorrelation values of the longevity indicators, we can state that the spatial distribution of the longevity indices in the dataset is spatially clustered more than would be expected if the underlying spatial processes were random, so we can reject the null hypothesis. Indeed, Moran’s I Z-score values indicate spatial autocorrelation at the 0.01 significance level for the Aging tendency, the 85+ ratio, and the 90+ ratio, and a 0.05 significance level for the Longevity index. No significance level occurs for the values of the Centenarian ratio and the Centenarity index. The results of the global spatial autocorrelation analysis are reported in Table 3.

**Figure 3:**
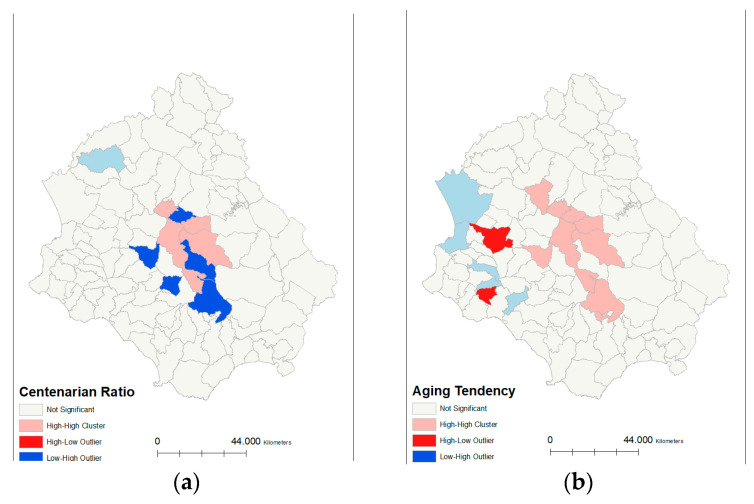

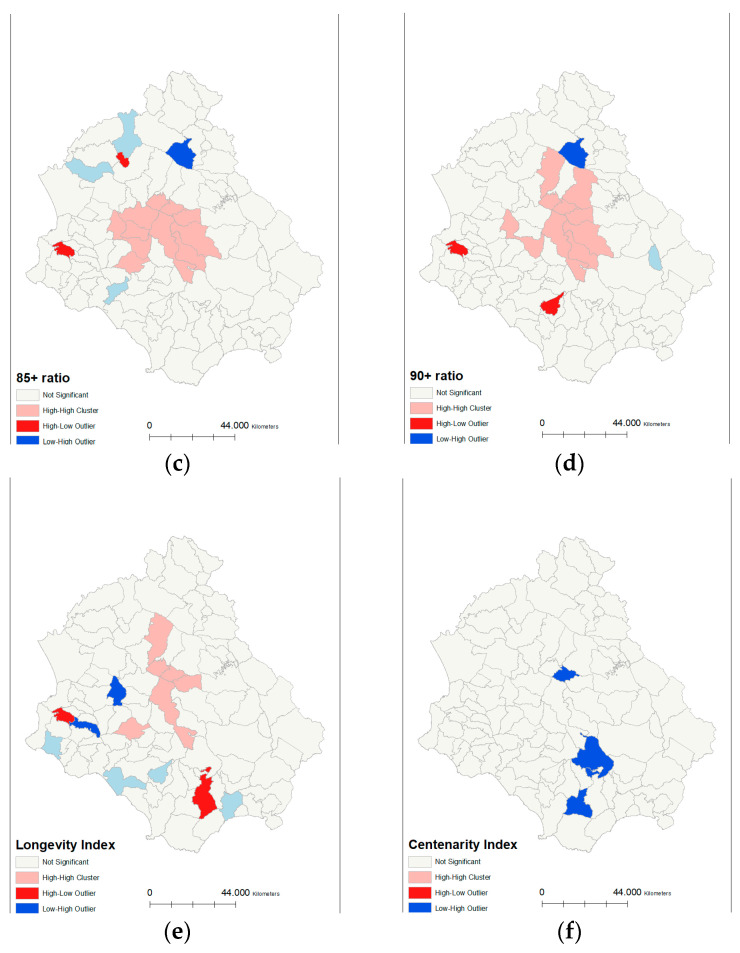
Local spatial distribution (Anselin local Moran’s I) maps of the cluster/outlier type for longevity indicators in Cilento municipalities. Note: (**a**) maps of the cluster/outlier type for Centenarian ratio; (**b**) maps of the cluster/outlier type for Aging tendency; (**c**) maps of the cluster/outlier type for 85+ ratio; (**d**) maps of the cluster/outlier type for 90+ ratio; (**e**) maps of the cluster/outlier type for Longevity index; (**f**) maps of the cluster/outlier type for Centenarity index.

Figure 3 shows the local spatial patterns (Anselin local Moran’s I) of longevity indicators in Cilento, Italy. The Centenarian ratio clusters were highlighted mainly in the designed area High-High and Low-Low, which are located in the central area of Cilento. Furthermore, due to the Aging tendency, the 85+ ratio, the 90+ ratio, and the Longevity index, the aggregated areas with a high longevity value defined High-High, were distributed mainly in the central and southeastern part of Cilento. The Low-Low areas were grouped into the Northwest and Southwest areas. In addition, the high and low areas were randomly placed in the areas surrounding the High-High and Low-Low regions. These results indicate that longevity is concentrated in the central and southeastern areas of Cilento and may be influenced by surrounding environmental factors.

### 3.2. The Spatial Pattern of Environmental Factors That May Influence the Distribution of Longevity

**Figure 4:**
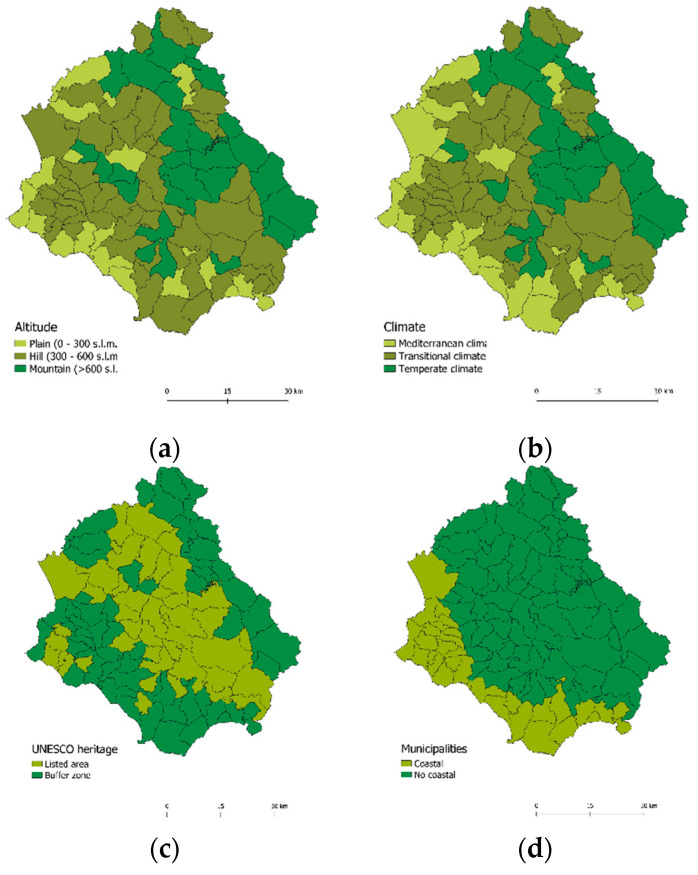
Spatial distribution in Cilento of altitude, climate, UNESCO heritage area, coastal/non-coastal areas. Note: (**a**) Altitude distributions of plains, hills, and mountains in Cilento; (**b**) Localization of Mediterranean, transitional, and temperate climate in Cilento; (**c**) Evidence of UNESCO heritage area and buffer zone; (**d**) Division between coastal and non-coastal areas in Cilento.

The spatial distribution of indicators relating to altitude, climate, and UNESCO heritage areas and coastal/non coastal areas, which may have an impact on the distribution of longevity in Cilento, is shown in Figure 4a–d, respectively.

In Figure 4a we can observe three different altitudes [47]: 1. the plain, located in the coastal area, between the towns: Pollica, Ascea, Pisciotta, San Mauro Cilento, Agropoli, Serre; 2. the hill, which covers most of the Cilento area; 3. the mountain, which is located in the northern and central-eastern area with Teggiano, Piaggine, Monte San Giacomo, Petrucci, Auletta, Caggiano. 

One of the most interesting features of Cilento is certainly the geomorphologic and climatic variability hardly found in other areas of the peninsula.

Figure 4b highlights the three climatic zones of Cilento: the Mediterranean climate, located in the coastal zone, with hot and dry summers and warm and humid winters, and an average temperature of 22–28 °C; the transitional climate, located in the central, central-western, and south-eastern hilly zone, with a temperate climate of transition to the Mediterranean and average temperatures around 20 °C; finally, the temperate climate, located towards the northern and central-eastern mountain zones, with cool summers and mild winters and average temperatures of 15–20 °C. The climatic variability of Cilento, which extends from the hottest and driest sectors to the coolest and most humid ones, is a source of exceptional value for the vegetation [48].

Figure 4c shows that Cilento also falls within the area of the Vallo di Diano National Park, which has been recognized as a UNESCO World Heritage Site for both the natural environmental and cultural value of the area. Therefore, the municipalities of Cilento are divided between the areas included in the UNESCO list and the buffer zones [50].

Finally, Figure 4d shows that the Cilento area is divided into 3/4 for the non-coastal zone and 1/4 for the coastal zone [47].

Analyzing the spatial pattern of environmental factors that may influence the distribution of longevity, we find that the Cilento areas with higher longevity tend to be located in the hinterland areas, at an altitude between 400 and 700 m.

#### 3.2.1. OLS Regression and GWR Models 

**Figure 5:**
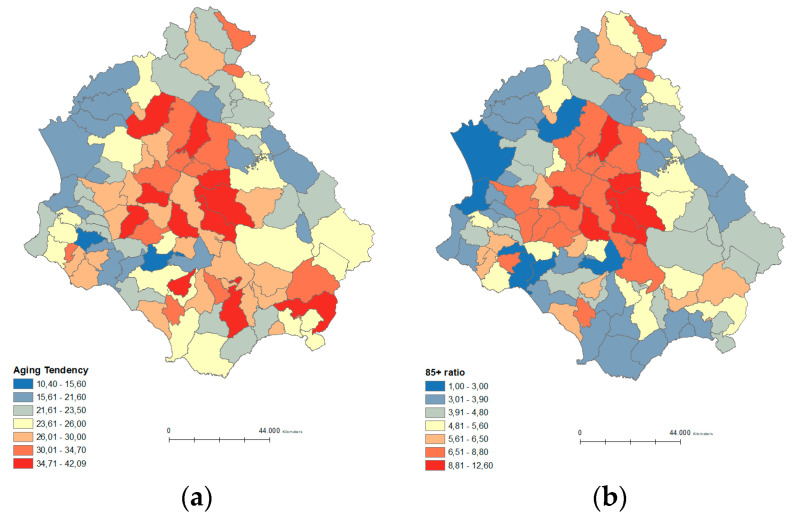

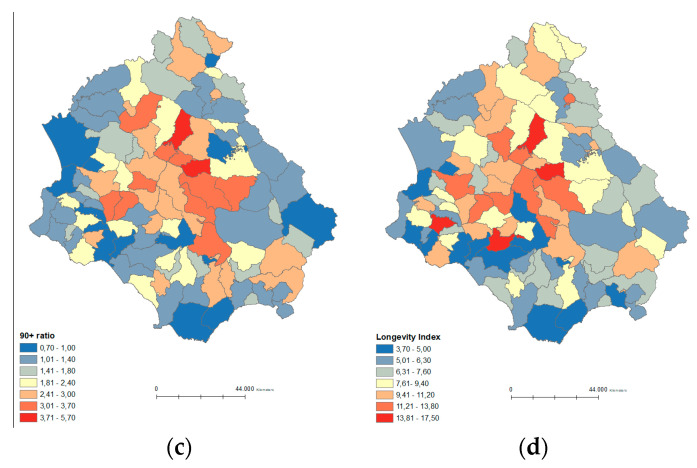
Results of the GWR models for Aging tendency, 85+ ratio, 90+ ratio and Longevity index showing the local R^2^. Note: (**a**) local R^2^ for Aging tendency; (**b**) local R^2^ for 85+ ratio; (**c**) local R^2^ for 90+ ratio; (**d**) local R^2^ for Longevity index.

OLS regression data and GWR models are reported in Table 4 and Figure 5, respectively. The OLS analysis revealed that longevity indicators in the studied area were dissimilarly influenced by different independent variables. In particular, a negative relationship was found between UNESCO buffer zones and Aging tendency, whereas a positive relationship was found between nonagenarians and the Cilento hinterland. Moreover, as show in Table 4, UNESCO buffer zones also show a negative relationship with the great old and nonagenarians, while the relationship between the longevity index with the Cilento hinterland and altitude emerged. 

In addition, to test for spatial autocorrelation, Moran’s I index was used to identify whether case densities were randomly distributed across the study area. We found that longevity densities were clustered across environmental factors, with altitude (Moran’s I = 0.26; z-score = 4.46; *p* = 0.0018), climate (Moran’s I = 0.21; z-score = 3.73; *p* = 0.0008), UNESCO heritage (Moran’s I = 0.27; z-score = 4.60; *p* = 0.0004), and littoral/non-littoral areas (Moran’s I = 0.65; z-score = 10.87; *p* < 0.0001). For the GWR models, shown in Figure 5, only variables that were significant in the OLS regression were used.

#### 3.2.2. Comparisons of the Performance between the OLS and GWR Models

Comparison of OLS regression and GWR models for all independent variables used in the analysis are shown in Table 5. 

### 3.3. Correlation between the Six Longevity Indicators

Analysis of the correlation between the six longevity indicators in Cilento, showed that nonagenarians (90+ ratio) have a strong correlation with the Longevity index and the Aging tendency. The great old (85+ ratio) have a correlation with the Longevity index, the Aging tendency and the 90+ ratio. The Centenarian ratio (C/10,000) correlates with the Aging tendency, the 85+ ratio, and the 90+ ratio, the Centenarity index, and the Longevity index. The Centenarity index correlates only with the Centenarian ratio (Table 6).

## 4. Discussion

By analyzing the association between six longevity indicators and five environmental factors, this study aimed to investigate the phenomenon of regional longevity in Cilento. Based on our results, we are able to support the hypothesis that healthy living and longevity in Cilento may be influenced directly or indirectly by several environmental factors, such as altitude, climate, UNESCO protected areas, and hinterland. In our opinion, there are at least three findings that support our hypothesis, which can be summarized as follows: 

First, it has been observed that longevity in Cilento is clearly manifested in central municipal areas of the territory and from there, by age groups, it spreads towards the southeastern area. This result, in agreement with the study of Deng et al., [59] confirms that there are only limited regions that show a high longevity index, and that the phenomenon of longevity is not universal. Herein, we found that centenarians are located in the heart of the clustered area at an altitude between 440 and 600 m above sea level (hilly area). Cilento area is characterized by a transitional climate between Mediterranean and temperate climate, with mild temperatures around 20 °C, fairly wet winters and moderate summer drought. The mild climate seems to be an important factor for the longevity phenomenon in Cilento. The results of our study agree with other studies [60,61] in confirming that the old, great old, nonagenarians and centenarians could benefit from the climate. From a clinical point of view, this could have important implications. Indeed, Mathieu et al., and Wyder [60,61], who mainly traced the genesis of medical interest in altitude climate, showed how mountain climate could play a role as therapeutic agent to reduce the risk of cardiovascular diseases. 

The second finding of our study is that the old, great old, and nonagenarians seem to be associated with the municipalities included in the UNESCO “World Heritage List”. A potential explanation could be, at least in part, found considering the diet [62] of those populations. In particular, the UNESCO natural area includes a Mediterranean park par excellence where grows a species of olive tree, Olea europaea. This food, with protected designation of origin, contains substances capable of playing an important antioxidant role [63], preventing neoplastic, inflammatory, cardiovascular, and metabolic disorders [64]. Pes et al. [65] demonstrated that a higher intake of olive oil has a beneficial effect on self-perceived health, physical performance, and sense organ function. Regarding Cilento area, Scelzo et al. [14] who studied nonagenarians and centenarians in Cilento, showed that those who lived in the hilly rural area of the territory adopted specific lifestyles, such as hard work, love of the land, family, and religion, which allowed them to maintain mental well-being and made them particularly resilient and optimistic. Moreover, the study by Pizza et al. [15] on Cilento nonagenarians also emphasized the importance of lifestyle, diet, but also individual personality factors. However, it is interesting to note that long-lived populations living in the buffer zone seem to be less protected. Therefore, more studies are necessary to explain this phenomenon.

As third finding, using a correlation analysis between the six longevity indicators, we found a linear continuity of the long-lived population in Cilento, which could ensure generational turnover among the old, the great old, the nonagenarians, and the current centenarians. Based on the results of the global autocorrelation values of the longevity indicators, we found that the spatial distribution of the longevity indices in the dataset resulted spatially clustered more than would be expected, when the underlying spatial processes were random. However, it should be underlined that no single longevity indicator was sufficient to assess regional longevity. In the current study, a complete picture of longevity in Cilento was obtained by using a set of indicators to delineate the different dimensions of longevity. 

Finally, all independent variables used in the analysis were compared using OLS regression and GWR models. Herein we found that longevity in Cilento is clearly manifested in the central municipalities of the territory and from there, by age group, it extends to the southeastern area. This result confirms that there are only limited regions that show a high longevity index, and that the longevity phenomenon is not universal [59]. Specifically, we found that longevous are negatively affected by UNESCO buffer zones, but positively affected by the hinterland areas of Cilento where UNESCO protected area municipalities are located. In addition, longevous benefited from the positive influence of altitude between 400 and 700 m. Climate seems to have no effect on longevity. Furthermore, in accordance with other studies, herein we found that GWR models achieved higher R^2^ and AICc values than the OLS regression model, indicating better performance of GWR models than OLS regression models [66,67,68].

Herein, longevity indicators in the studied area were dissimilarly influenced by several independent variables. In particular, we found a negative relationship between UNESCO buffer zones and Aging tendency, while a positive relationship between nonagenarians and the Cilento hinterland. Furthermore, UNESCO buffer zones also show a negative relationship with the great old and nonagenarians, while a relationship between the longevity index with the Cilento hinterland and altitude emerged. This result supports our hypothesis that countries included in the UNESCO heritage list represent a possible natural protective factor for longevity. 

From a political and economic point of view, considering that an aging society implies an increase in health care spending, it should be mandatory to consider more and more interventions to support healthy aging, selecting those factors that are able to ward off disease and make long-term care policies economically sustainable. 

This study has some limitations. First, it analyzed longevity in Cilento by considering only a specific year without examining a longer period of time. Second, it investigates the phenomenon of longevity in Cilento by analyzing only specific environmental factors, such as altitude, climate, UNESCO-protected areas, and hinterland, that could directly or indirectly influence the extent of healthy living and long life in Cilento, but did not consider factors such as drinking water that might have traces of elements beneficial to human health. Third, despite that some environmental factors could be better studied by techniques, such as remote sensing or land-use data, in the present study the input layers for environmental factors were not studied. 

## 5. Conclusions

To the best of our knowledge, this is the first study to define the area of longevity in Cilento through six longevity indicators and four environmental factors. The results obtained from the study showed that environmental factors, such as hill altitude, transitional climate between Mediterranean and temperate climate, UNESCO heritage areas, and hinterland areas, can contribute directly or indirectly to local longevity in Cilento and centenarians. Further studies are needed to better understand the relationship between health, diet, and living area. In addition, studies related to the association between environmental and genetic factors on local longevity in Cilento should be encouraged and investigated to understand what factors are able to modify the epigenome to establish healthy aging. 

## Figures and Tables

**Table 1:** Information of the dependent variables.

Indicators	Definition	Description	Bibliographic References
Centenarian ratio	Number of centenarians per 10,000 population	To reflect the extreme regional longevity rate	Song et al., 2016 [8]
Aging tendency	65+ young old population/total population	To reflect the total local old population and the aging trend	Wang et al., 2015 [6]
85+ ratio	85+ great old population/total population	To reflect the proportion of the great old in the total population	Lv et al., 2011 [7]
90+ ratio	90+ nonagenarian population/total population	To reflect the proportion of nonagenarians in the total population	Lv et al., 2011 [7]
Longevity index	The ratio of the 90+/65+ population	To reflect longevity among the old population	Magnolfi et al., 2007 [9]
Centenarity index	The ratio of centenarians to the 90+ population	The ratio of centenarians to the 90+ population	Magnolfi et al., 2007 [9] Lv et al., 2011 [7]

Note: + = more than.

**Table 2:** Information of the independent variables.

Environmental Indicators		Description of Variables
Altitude	Elevation	Plain 0–300 m Hill 300–600 m Mountain > 600 m
Climate	Mean temperature; annual precipitation	Mediterranean climate Temperate transitional climate
UNESCO heritage	Elevation	Listed area (presence in the list of UNESCO municipalities) Buffer zone (absence in the list of UNESCO municipalities)
Municipalities	Elevation	Coastal (presence of municipalities in the coastal zone) No coastal (absence of municipalities in the no coastal zone)

Note: The table shows the subsets of the different indicators.

**Table 3:** Synthesis of global Moran’s I indices.

Longevity Index	Global Moran’s I Index	Z-Score	*p*-Value
Centenarian ratio	0.08	1.64	0.099
Aging tendency	0.21	3.89	0.001 **
85+ ratio	0.35	6.23	<0.001 **
90+ ratio	0.22	4.12	0.003 **
Longevity index	0.11	2.21	0.026 *
Centenarity index	−0.04	−0.60	0.544

Note: * denotes *p* < 0.05; ** denotes *p* < 0.01, indicates the statistical significance of the Moran’s test; z-score indicate statistical significance of clustering (it must be greater than 1.96 at *p* < 0.05 and greater than 2.58 at *p* < 0.01; *p* value probability of error in rejecting the null hypothesis.)

**Table 4:** Results of OLS regression and values of the Variance inflation factor (VIF).

Longevity Indicators	Environmental Variables	Coefficient	t	*p* Value	VIF
Centenarian Ratio		There are no statistically significant variables			
	Climate (temperate-transitional)	0.08	0.09	0.92	1.31
Model 1. Aging tendency	UNESCO buffer zone	−4.60	−4.18	0.006	1.08
	Non coastal area	1.37	1.06	0.29	1.27
	Climate (temperate-transitional)	0.24	0.78	0.43	1.31
Model 2. 85+ ratio	UNESCO buffer zone	−1.33	−3.46	0.008	1.08
	Non coastal area	0.88	1.95	0.05	1.27
	Altitude (hill-mountain)	0.09	0.06	0.94	1.26
Model 3. 90+ ratio	UNESCO buffer zone	−0.60	−3.39	0.010	1.09
	Non coastal area	0.51	2.49	0.014	1.21
	Altitude (hill-mountain)	0.73	2.68	0.009	1.87
Model 4. Longevity index	UNESCO buffer zone	−0.98	−1.73	0.08	1.03
	Non coastal area	1.56	2.53	0.012	1.03
Centenarity Index		There are no statistically significant variables			

Note: Significance *p* value < 0.05; VIF: variance inflation factor.

**Table 5:** Performance comparison between OLS regression and GWR models.

	OLS Regression		GWR Models	
Parameters	R^2^	AICc	R^2^	AICc
Model 1	0.18	642.25	0.23	643.76
Model 2	0.20	426.12	0.25	426.22
Model 3	0.19	267.37	0.25	266.97
Model 4	0.10	508.44	0.21	504.26

Note: Models 1–4 indicate the models analyzed with OLS regression in Table 4; R^2^ indicates R-Squared, coefficient of determination; AICc—Akaike’s Information Criterion.

**Table 6:** Correlation analysis among the six longevity indicators.

	Centenarian Ratio	Aging Tendency	85+ Ratio	90+ Ratio	Centenarity Index	Longevity Index
Centenarian ratio	1.000					
Aging tendency	0.335 *	1.000				
85+ ratio	0.456 **	0.713 **	1.000			
90+ ratio	0.366 **	0.724 **	0.793 **	1.000		
Centenarity index	0.852 **	0.119	0.134	0.054	1.000	
Longevity index	0.292 *	0.275 *	0.610 **	0.758 **	0.009	1.000

Note: * significant correlation with *p* value ≤ 0.05; ** significant correlation with *p* value 0.01.

## Data Availability

The data included in this manuscript were provided by the National Institute of Statistics and the Cilento Municipal Archives. Therefore, we are not authorized to share the data with third party organizations. However, the corresponding author is available to provide any explanation to the Editor if requested.
